# Interactive Effects of Chemical Pretreatment and Drying on the Physicochemical Properties of Cassava Flour Using Response Surface Methodology

**DOI:** 10.1155/2020/7234372

**Published:** 2020-09-25

**Authors:** Elohor Oghenechavwuko Udoro, Tonna Ashim Anyasi, Afam I. O. Jideani

**Affiliations:** ^1^Department of Food Science and Technology, School of Agriculture, University of Venda, Thohoyandou, 0950 Limpopo Province, South Africa; ^2^Department of Food Technology, School of Industrial Technology, Akanu Ibiam Federal Polytechnic, P.M.B. 1007, Afikpo-Unwana, Ebonyi State, Nigeria; ^3^Department of Food Science and Technology, Cape Peninsula University of Technology, P.O. Box 1906, Bellville 7535, South Africa

## Abstract

Calcium chloride and citric acid (0.6–3.4%*w*/*v*) were separately applied in the pretreatment of two South African cassava landraces (white and red) processed into flour at drying temperatures of 45–74°C. Optimisation using the response surface methodology showed ash (0.79–4.42%) and crude fibre (2.77–5.12%) increased as the drying temperature (DT) and concentration of pretreatment (COP) increased. Starch content (78.06–84.71%) was not influenced by the processing variables. Both pretreatments improved the lightness and whiteness index of cassava flour. Optimal processing conditions of 70°C DT and 3%*w*/*v* COP were the same for the proximate composition of cassava flour from all experimental groups.

## 1. Introduction

The world's most important staple root crop is cassava (*Manihot esculenta* Crantz), which is also known as tapioca or manioc [1]. It is a woody shrub belonging to the family *Euphorbiaceae*. The tuberous roots, rich in starch, are the main storage organ in cassava and the major part of the plant that is mostly consumed [2, 3]. Approximately one billion people rely on cassava for food daily, predominantly in the tropical and subtropical regions of Latin America, Asia, and Africa [4]. Cassava flour is a major product of the root that has found various applications for both domestic and industrial use. The flour is a dry powdery product obtained from the root with simple process technology [5].

Cassava root handling and processing are faced with a challenge of rapid postharvest physiological deterioration (PPD) [6, 7]. Rapid PPD is a complex physiological and biochemical process that begins with vascular streaking characterised by blue-black discoloration. The blue-black discoloration process is caused by microbial activity leading to a total deterioration of the root [8, 9]. The use of chemicals regarded as safe, such as calcium chloride, citric acid, and ascorbic acid, in food processing to control enzymatic browning and discoloration is a common practice. There is available literature on the use of ascorbic acid and citric acid in the treatment of white yam flour [10]; the use of sulphite, calcium chloride, and citric acid in the treatment of potato flour [11, 12]; and the use of calcium chloride treatment for the control of enzymatic browning of minimally processed cassava chips [13, 14]. However, there is sparse literature on the use of safe chemical pretreatment in the processing of cassava flour.

Processing of cassava flour involves several units which can be varied as the quality of flour is determined by the process it undergoes [15]. Response surface methodology (RSM) is a mathematical and statistical technique used for defining the effect of more than one processing variable on a response (parameter) of interest. Pornpraipech et al. [16] used RSM to determine the drying behaviour of cassava chips using two cutting shapes (rectangular and circular) evaluated under different temperatures (60, 80, 100, and 120°C). The effect of drying temperature and time on the thermal and physical properties of cassava flour was investigated by Omolola et al. [17]. Nemaungani et al. [18] in their preliminary findings showed the improved colour quality of solar-dried cassava flour as a result of calcium chloride and citric acid pretreatment. Presently, there is no information on the interactive effects of chemical pretreatment and drying temperature on cassava flour using RSM. Therefore, in this research, citric acid and calcium chloride (0.6–3.4%*w*/*v*) are separately applied in the pretreatment of flour from two South African cassava landraces (white and red) under drying temperatures of 45–74°C. RSM is employed to determine the linear, interactive, and quadratic effects of these processing variables on the colour properties (*L*^∗^, *a*^∗^, *b*^∗^, chroma, colour difference, whiteness index, and brownness index) and proximate components (starch, ash, moisture, and fibre content) of cassava flour.

## 2. Materials and Methods

### 2.1. Sourcing and Preparation of Cassava Root

Two landraces of cassava (white and red) were sourced from the Institute of Tropical and Subtropical Crops—Agricultural Research Council (ITSC-ARC), Levubu, Limpopo Province, South Africa (22.946°S, 30.485°E). The roots were harvested fourteen months after planting and sorted before washing with tap water generously to remove adhering dirt and avoid contamination during processing. Within 24 h after harvest, processing commenced after ascertaining that the roots were in a fresh state, before the onset of deterioration [14].

### 2.2. Design of Experiment

Before the conduct of the experiment, Design Expert (DE) software version 11 generated the experimental conditions for processing the flour samples. The conduct of the experiment was done using the central composite design with two independent variables: concentration of pretreatment (COP) and drying temperature (DT).  Table 1 shows the levels of the independent variables. Two pretreatments, citric acid (CA) and calcium chloride (CC), were used separately with the same variation in DT giving rise to four experimental groups. The four experimental groups are as follows: citric acid pretreated flour from the red landrace (CAR), calcium chloride pretreated flour from the red landrace (CCR), citric acid pretreated flour from the white landrace (CAW), and calcium chloride pretreated flour from the white landrace (CCW). The dependent variables are the proximate components (starch, ash, moisture, and fibre content) and colour properties (*L*^∗^, *a*^∗^, *b*^∗^, chroma, colour difference, whiteness index, and brownness index) of cassava flour. Each experimental run generated by the DE software was done in triplicate.

### 2.3. Processing of Pretreated Cassava Flour

**Figure 1:**
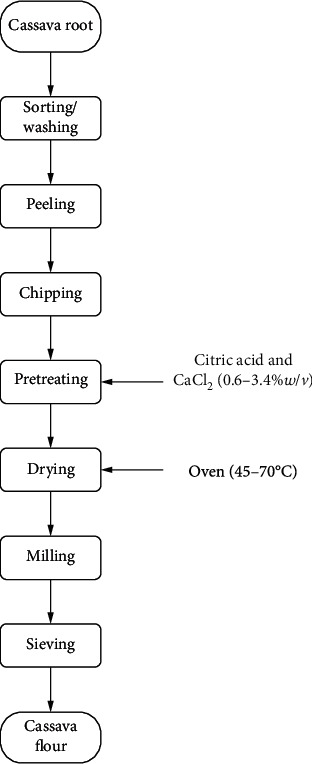
Process flow chart for pretreated cassava flour.

The method described by the Federal Institute of Industrial Research [19] was employed, with modifications, in processing the flour. Modifications involved the application of different concentrations of chemical pretreatment and drying temperatures (Figure 1) for each experimental sample as generated by the central composite design. The washed roots were manually peeled and cut into small slices with a knife. Fresh cassava root slices (1 ± 0.5 cm thickness) were pretreated by steeping them in the chemical solution for about 20 min after which they were dried in an oven (Ecotherm, 240L digital oven, South Africa) at different temperatures (45–74°C) for 24 h. Milling of dried chips was done with the aid of a dry milling machine (Polymix PX-MFC 90D, Switzerland). Flour obtained from the miller was subjected to sieving through a 500 *μ*m sized aperture sieve and stored, till further analysis, in sample bags made of paper material at room temperature. Two control samples were processed, one from each landrace, using the same procedure as the experimental samples save for pretreatment and DT was 60°C. The control samples served as reference samples to experimental samples during statistical analysis within a landrace.

### 2.4. Determination of Colour Properties of Cassava Flour

A colorimeter (ColorFlex, HunterLab, USA) was employed in the determination of the following colour properties of the samples: *L*^∗^ (lightness/darkness), *a*^∗^ (redness/greenness), and *b*^∗^ (yellowness/blueness). The colorimeter was calibrated with a standard white (*L*^∗^ = 93.71, *a*^∗^ = –0.84, and *b*^∗^ = 1.83) and black plate before use. Chroma, colour difference (Δ*E*), whiteness index (WI), and brownness index (BI) were calculated as follows [20]:

(1) $$\begin{array}{c} \text{Chroma}=\sqrt{a^{*2}+b^{*2}}, \end{array}$$

(2) $$\begin{array}{c} \Delta E=\sqrt{{\left(L0-L^{*}\right)}^{2}+}{\left(a0-a^{*}\right)}^{2}+{\left(b0-b^{*}\right)}^{2}, \end{array}$$

(3) $$\begin{array}{c} \text{WI}=\sqrt{{\left(100-L^{*}\right)}^{2}+a^{*2}+b^{*2}}, \end{array}$$

(4) $$\begin{array}{c} \text{BI}=100\times \frac{x-0.31}{0.71}, \end{array}$$

where *x* = (*a*^∗^ + 1.75*L*^∗^)/(5.645*L*^∗^ + *a*^∗^ − 3.012*b*^∗^).

### 2.5. Near-Infrared Spectroscopy (NIRS) Analysis of Cassava Flour

Percentage ash, crude fibre, moisture, and starch contents of the samples were measured using an NIR analyser (DA 7250 Perten Instruments, Sweden). The analyser's open-faced sample dish was filled with cassava flour and placed in the NIR analyser. The samples were scanned by infrared rays emitted by the machine, and the software on the PC displayed the readings obtained. The NIR analyser was previously standardised by a selection of calibration and validation samples and reference data obtained by routine laboratory analysis [21].

### 2.6. Statistical Analysis and Optimisation

Design Expert software version 11 was employed in the analysis of variance (ANOVA) of the linear, interactive, and quadratic effects of the model parameters on cassava flour properties. Regression models, coefficient of determination (*R*^2^), *p* values, *F* values, response surface plots, contour plots, and optimisation conditions were generated by the software for all four experimental groups. Statistical data analysis was done with the IBM SPSS Statistics software version 25 (IBM Corp., NY, USA) to ascertain the significant difference between means of the experimental and reference/control samples within each experimental group at a 95% confidence level. One-way ANOVA and separation of means were done using Duncan's multiple range test.

## 3. Results and Discussion

### 3.1. Effect of Chemical Pretreatment and Drying Temperature on the Ash Content of Cassava Flour

**Figure 2:**
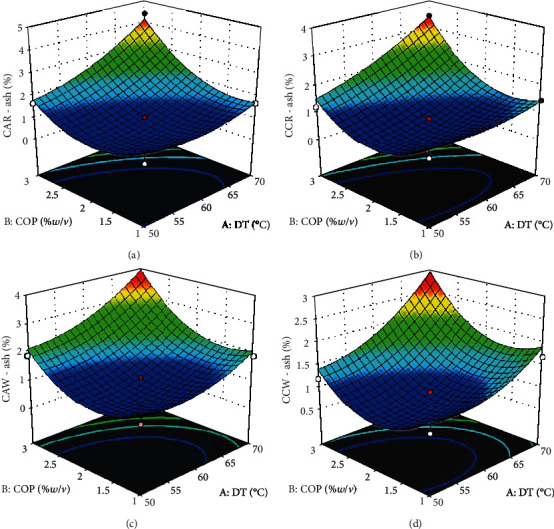
Response surface plots for ash content of cassava flour. (a) Cassava from red landrace pretreated with citric acid. (b) Cassava from red landrace pretreated with calcium chloride. (c) Cassava from white landrace pretreated with citric acid. (d) Cassava from white landrace pretreated with calcium chloride. DT—drying temperature (°C); COP—concentration of pretreatment (%*w*/*v*).

The ash content of cassava is an indication of its nonvolatile content and mineral richness [3]. All four experimental groups CAR, CCR, CAW, and CCW had similar ash contents within the range of 0.79 to 4.42%, and they varied significantly within the groups. Significance in variation implies that the processing conditions affected the ash content of flour. Some values of ash content obtained in this study are similar while others were above the range (0.33–1.04%) reported by Ukenye et al. [22] for cassava root. The values (0.74–1.43%) reported by Aniedu and Omodamiro [23] for cassava flour are within the range obtained in this study. Response surface plots for the ash contents of all four experimental groups had a similar shape (Figure 2) in which the ash contents increased with the concentration of pretreatment and drying temperature. The findings in this study disagree with those of Montagnac et al. [3] who stated that cassava processing significantly reduces the ash content of the roots and Ayetigbo et al. [24] who postulated that severe processing involving the application of high temperatures and chemicals may significantly decrease ash in cassava diets. The increase in ash content as COP increased could be attributed to elements from pretreatment solutions used in soaking fresh cassava chips in water, before drying, which may be retained in the samples after processing. This observation was buttressed by ANOVA of the model parameters *F* and *p* values in Table 2 which indicate that the linear, interactive, and quadratic effects of the experimental factors significantly influenced the ash content of cassava flour. Regression models generated by RSM (Table 3) and the coefficient of determination (*R*^2^) of the ash content were relatively high between 0.9446 and 0.9710, which show that the model fits.

### 3.2. Effect of Chemical Pretreatment and Drying Temperature on the Moisture Content of Cassava Flour

**Figure 3:**
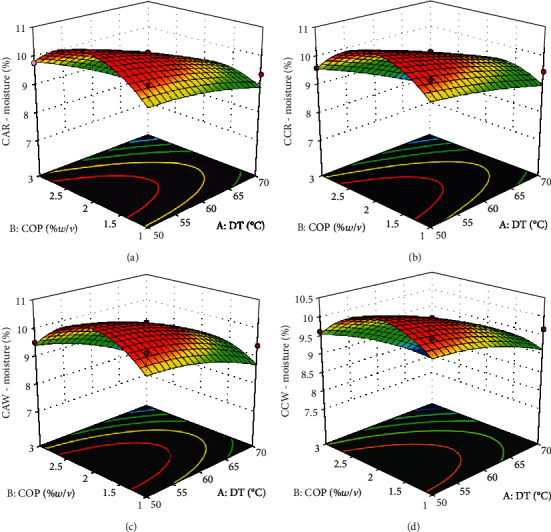
Response surface plots for percentage moisture of cassava flour. (a) Cassava from red landrace pretreated with citric acid. (b) Cassava from red landrace pretreated with calcium chloride. (c) Cassava from white landrace pretreated with citric acid. (d) Cassava from white landrace pretreated with calcium chloride. DT—drying temperature (°C); COP—concentration of pretreatment (%*w*/*v*).

High moisture content in foods aids the growth of microbes [25]; hence, drying is applied to eliminate moisture. The cassava flour samples processed under varying conditions had moisture contents between 7.43 and 10.50%. The values in this study are within the percentage moisture range of 3.59 to 11.53 reported by Onitilo et al. [26] for flour from white- and yellow-fleshed cassava roots. The moisture content of the yellow-fleshed cassava root was higher than the white-fleshed roots. This was not the case in this study as the response surface plots of the moisture content of all experimental groups displayed the same pattern (Figure 3). The moisture contents of the samples increased and decreased along the *X*2 (COP) axis. A slight decrease in moisture was observed with an increase along the *X*1 (DT) axis. ANOVA of model parameters produced *p* values less than 0.05 for the linear effect of DT and quadratic effect of COP. Regression models yielded a coefficient of variation of 0.8631–0.8990, and all had positive intercepts. Materials, like flour, having above 12.5% moisture possess less storage stability than those with less moisture content; hence, a moisture content of not more than 12.5% is generally specified for flours [27]. The amount of moisture contained in all the samples is below the 12.5% moisture level specified for flours. Therefore, it can be deduced that the pretreatment and drying temperature applied in this study are suitable to produce cassava flour with storage stability and potential for prolonged shelf life.

### 3.3. Effect of Chemical Pretreatment and Drying Temperature on the Fibre Content of Cassava Flour

**Figure 4:**
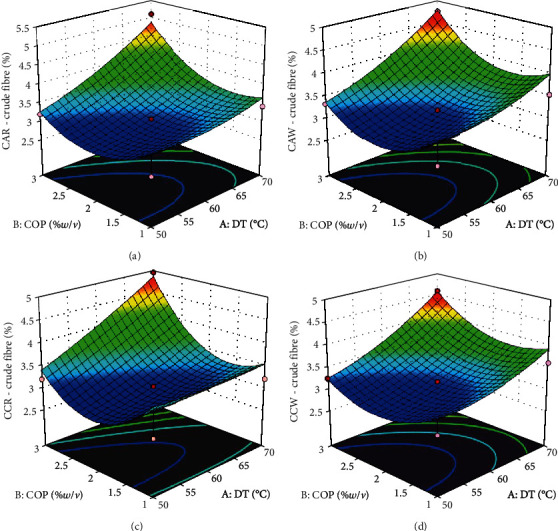
Response surface plots for crude fibre content of cassava flour. (a) Cassava from red landrace pretreated with citric acid. (b) Cassava from white landrace pretreated with citric acid. (c) Cassava from red landrace pretreated with calcium chloride. (d) Cassava from white landrace pretreated with calcium chloride. DT—drying temperature (°C); COP—concentration of pretreatment (%*w*/*v*).

Textural behaviour and in vitro digestibility of cassava flour are influenced by its residual fibre [24]. The crude fibre content was between 2.77 and 5.12%. The least and highest crude fibre contents were exhibited by samples of experimental runs 1 (50°C/1%*w*/*v*) and 4 (70°C/3%*w*/*v*) in all the experimental groups. It gives a clue that the crude fibre content increased with DT and COP. The response surface plots for all experimental groups (Figure 4) align with this observation. Aniedu and Omodamiro [23] reported lower values (0.62–1.63%) for flour processed from white-flesh and yellow-flesh cassava varieties. The authors reported that the fibre content in cassava flour from the white-flesh variety is generally higher than that of the yellow-flesh variety. Ukenye et al. [22] also reported relatively higher fibre content (0.62–4.92%) in the roots of white-flesh cassava than in those of the yellow-flesh varieties. Differences in fibre content maybe attributed to differences in varietal composition and age of harvest [3]. The process flow in this study did not involve the removal of fibre from the roots (Figure 1) as is done in starch extraction and in some cassava flour processing methods. The omission of this step (removal of fibre) could be responsible for the relatively high fibre content in these samples. ANOVA of model parameters shows that the linear effect of drying temperature and the quadratic effect of concentration of pretreatment significantly influenced (*p* < 0.05230) the fibre content of the flours. The high fibre content obtained in this study may positively influence the dietary fibre content available in the flours. Consumption of an ample quantity of dietary fibre decreases the risk of diseases such as obesity, constipation, gallstones, diabetes, and coronary heart diseases [28]. This is an indication that the landraces and processing conditions are suitable for producing cassava flour advantageous to the health of consumers.

### 3.4. Effect of Chemical Pretreatment and Drying Temperature on the Starch Content of Cassava Flour

**Figure 5:**
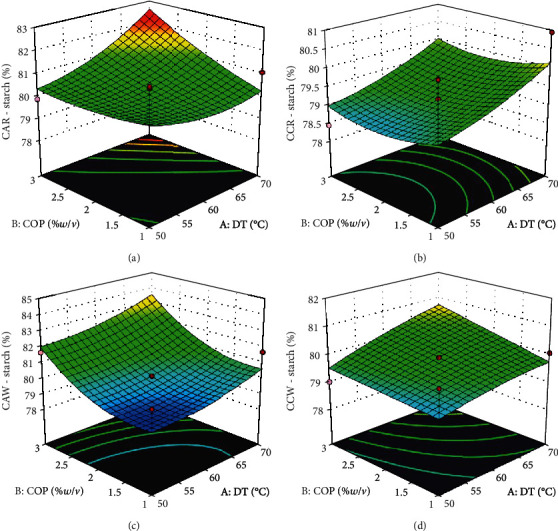
Response surface plots for starch content of cassava flour. (a) Cassava from red landrace pretreated with citric acid. (b) Cassava from red landrace pretreated with calcium chloride. (c) Cassava from white landrace pretreated with citric acid. (d) Cassava from white landrace pretreated with calcium chloride. DT—drying temperature (°C); COP—concentration of pretreatment (%*w*/*v*).

The percentage starch composition of 78.06–84.71 was observed with significant variation (*p* < 0.05) across all experimental groups. The *F* values (<0.01–15.15) in Table 2 and the coefficient of determination (0.2120–0.7792) in Table 3 were relatively low compared to other proximate components of the flour. The ANOVA of model parameters produced *p* values below 0.05 for the model and linear effect of COP for samples pretreated with citric acid from the white landrace (CAW). This is evident in the response surface plots (Figure 5) of CAW, the *R*^2^ of 0.7792, and the *F* value of 15.15 which was higher than all other experimental groups. All other *p* values were above 0.05 indicating that the model did not fit, while the linear, interactive, and quadratic effects of the processing variables did not have a significant effect on the starch content of the flours.

### 3.5. Effect of Chemical Pretreatment and Drying Temperature on the Colour Properties of Cassava Flour

Colour is a physical yet vital parameter that determines consumers' preference for a product. Tables 4 and 5 are results of ANOVA between means of the colour properties of cassava flour pretreated with calcium chloride. ANOVA reveals that processing cassava flour under varying COP and DT produced samples with significantly variant (*p* < 0.05) *L*^∗^, *a*^∗^, *b*^∗^, chroma, whiteness index (WI), brownness index (BI), and change in colour (Δ*E*). Regression models relating the colour properties to actual levels of processing conditions are depicted in Table 6. The coefficient of determination was between 0.4241 (Δ*E*—CAR) and 0.9981 (*b*^∗^—CCW).

*L* ^∗^ values of the cassava flour samples were between 91.37 and 93.65 with the control sample significantly lower (*p* < 0.05) than the experimental samples in all four groups. This shows that pretreatment of cassava flour from both landraces with citric acid or calcium chloride enhances the lightness of the flour. Soaking and grating were reported to cause a significant (*p* < 0.05) increase in the *L*^∗^ values of cassava flour [29]. The significance of the quadratic effect of COP on the *L*^∗^ values (not reported) in all experimental groups and the increase of *L*^∗^ values along the COP axis of the response contour plots (not reported) buttresses the fact that pretreatment improved the lightness of the flour. These findings are in line with the report of Nemaungani et al. [18] on citric acid and calcium chloride pretreated flour processed from solar-dried cassava chips.

The measure of redness/greenness (*a*^∗^ value) and blueness/yellowness (*b*^∗^ value) of the flour samples ranged from -0.52 to 0.97 and from 4.55 to 8.24, respectively. Hasmadi et al. [30] reported similar *a*^∗^ values (-0.11 and 0.63) and *b*^∗^ values (4.55 and 8.24) for flour processed from two cassava varieties grown in Sabah, Malaysia. The ranges of *a*^∗^ (-1.75 to -0.74) and *b*^∗^ (6.42 to 9.92) values reported by Chiwona-Karltun et al. [29] for cassava flour subjected to different cassava genotypes are also similar to the values obtained in this study. Analysis of variance shows that the *a*^∗^ values of the control sample in citric acid pretreated flours were significantly lower (*p* < 0.05) than the experimental samples within the respective groups (Tables 7 and 8). Control samples CCR and CCW exhibited significantly higher *b*^∗^ values, chroma, and browning index within their respective experimental groups. This implies that calcium chloride may have a reducing effect on the *b*^∗^ value, chroma, and browning index of the flours.

Change in colour (Δ*E*) is a function of the difference between the *L*^∗^, *a*^∗^, and *b*^∗^ values of the experimental samples and the control. Colour change was the highest (9.54–9.69) in cassava flour processed from the white landrace pretreated with citric acid (CAW) and the lowest (1.11–1.81) in cassava flour processed from the red landrace pretreated with citric acid (CAR). This gives a hint on the effect of landrace on the colour properties of cassava flour. As for the whiteness index (WI), the trend was similar to that of the *L*^∗^ value. The control in all experimental groups had the least WI. The values of *L*^∗^ (87.67–93.57), *a*^∗^ (-0.27–1.1), *b*^∗^ (8.4–11.83), chroma (8.4–11.87), and WI (82.88–89.42) reported in the work of Omolola et al. [17] for cassava flour are similar to those in this study. The findings of Falade and Ayetigbo [31] on the effect of citric acid modification on colour properties of yam starch agree with the trend in this study. Falade and Ayetigbo [31] reported that *L*^∗^, *a*^∗^, and WI increased while *b*^∗^ values decreased with citric acid modification of yam starches from four different cultivars.

### 3.6. Multiresponse Optimisation of Cassava Flour Processing

**Figure 6:**
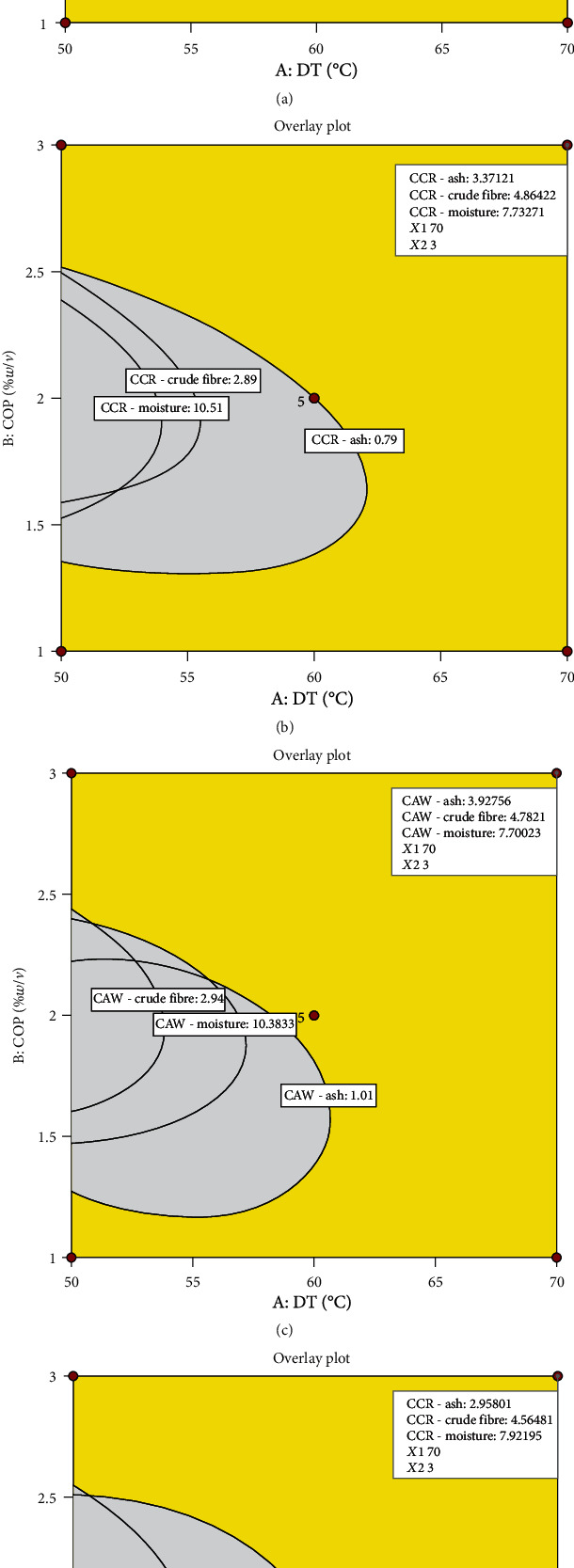
Multiresponse optimisation overlay plots of processing conditions and proximate components of cassava flour. (a) Cassava from red landrace pretreated with citric acid. (b) Cassava from red landrace pretreated with calcium chloride. (c) Cassava from white landrace pretreated with citric acid. (d) Cassava from white landrace pretreated with calcium chloride. DT—drying temperature (°C); COP—concentration of pretreatment (%*w*/*v*).

Multiresponse optimisation of proximate components and colour properties of cassava flour was conducted, separately, using the Design Expert software. The following optimisation goal for the former was set: starch content in range, minimised moisture content, and maximised ash and crude fibre contents. Overlay plots for optimal processing conditions for cassava flour from all four experimental groups with the desired qualities are shown in Figure 6. Optimal conditions (70°C/3%*w*/*v*) for all four experimental samples were the same. However, the responses and desirability differed for CAR (ash—4.17; crude fibre—4.88; moisture—7.70%; and desirability—0.912), CCR (ash—3.37; crude fibre—4.86; moisture—7.73; and desirability—0.955), CAW (ash—3.93; crude fibre—4.78; moisture—7.70; and desirability—0.992), and CCW (ash—2.96; crude fibre—4.57; moisture—7.92; and desirability—0.997).

**Figure 7:**
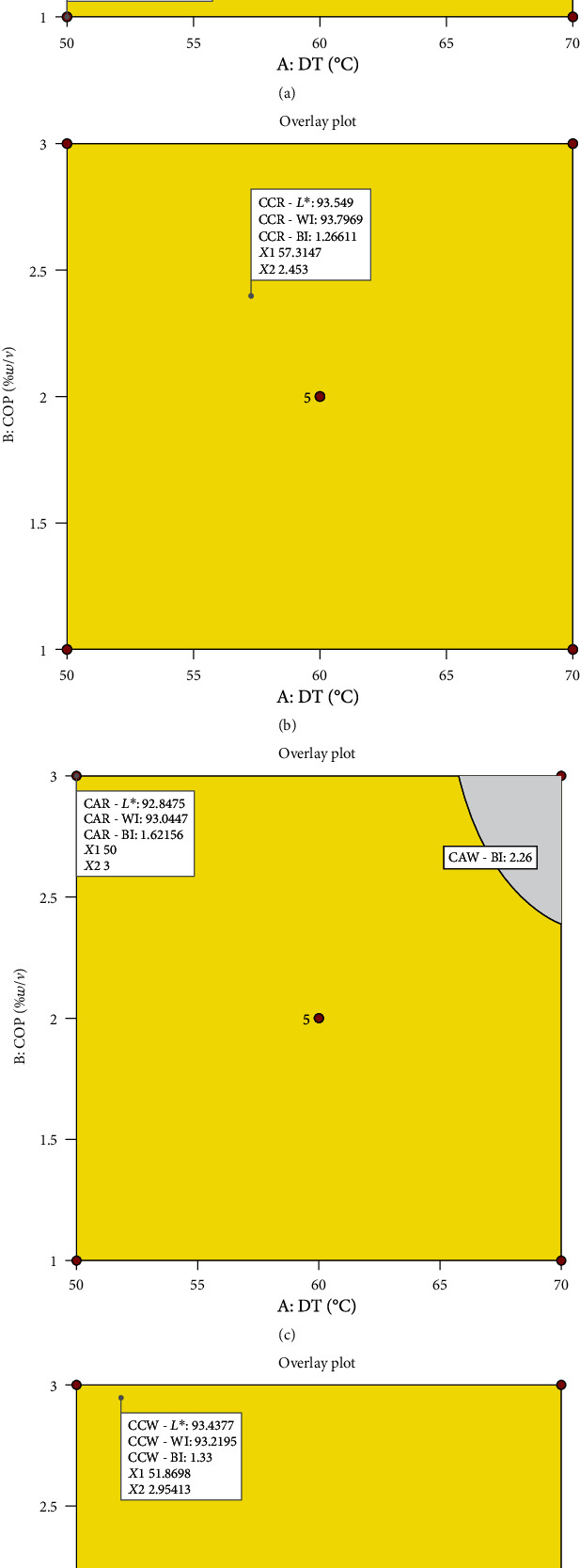
Multiresponse optimisation overlay plots for optimisation of processing conditions and colour properties of cassava flour. (a) Cassava from red landrace pretreated with citric acid. (b) Cassava from red landrace pretreated with calcium chloride. (c) Cassava from white landrace pretreated with citric acid. (d) Cassava from white landrace pretreated with calcium chloride. DT—drying temperature (°C); COP—concentration of pretreatment (%*w*/*v*).

Optimisation of colour properties had the following targets: minimised BI and maximised WI and *L*^∗^ values. The two maximised colour parameters correspond to consumers' preference for cassava flour. Omolola et al. [17] stated that whiteness is the characteristic and acceptable colour property associated with cassava flour by consumers. The RSM overlay plots generated from the Design Expert software (Figure 7) show the processing conditions for obtaining cassava flour with these desired properties for all four experimental groups. The optimal processing conditions are as follows: CAR—50°C/1%*w*/*v* and 0.795 desirability; CCR—57.31°C/2.45%*w*/*v* and 0.546 desirability; CAW—50°C/3%*w*/*v* with desirability of 0.846; and CCW—51.87°C/2.95%*w*/*v* and 0.821 desirability. Low *a*^∗^ and *b*^∗^ values with relatively high *L*^∗^ and WI values recorded for all flour samples indicate that the samples are white. Whiteness is an advantageous consumer property especially for substitution of wheat flour.

## 4. Conclusion

Application of response surface methodology was effective in investigating the linear, quadratic, and interactive effects of calcium chloride or citric acid pretreatment (0.6–3.4%*w*/*v*) and drying temperature (45–74°C) in cassava flour processing. The study showed that the processing conditions yielded cassava flours with good shelf stability, increased ash content, and high fibre content which is good for the health of consumers. An increase in lightness and whiteness index of the flour indicates that both calcium chloride and citric acid pretreatment has mitigating efficacy against enzymatic browning associated with PPD and processing of cassava flour. The chemical pretreatment and drying temperatures had no significant effect (*p* > 0.05) on the starch content of cassava flours. Response surface plots, multiresponse overlay optimisation plots, and regression models generated from this study assist in eliminating the preexisting gap of the interactive effect of pretreatment and drying temperature on proximate composition and colour properties of cassava flour.

## Figures and Tables

**Table 1:** Levels of independent variables used for central composite design.

Pretreatment	Code	DT (°C)	COP (%*w*/*v*)
Citric acid	-1	50	1
	0	60	2
	1	70	3
Calcium chloride	-1	50	1
	0	60	2
	1	70	3

-1 = upper limit; 0 = central point; 1 = lower limit; DT—drying temperature; COP—concentration of pretreatment. Note: two pretreatments (citric acid and calcium chloride) were used separately.

**Table 2:** ANOVA of the effect of model parameters on proximate components of cassava flour.

Source	Ash		Crude fibre		Moisture		Starch	
	**F** value	**p** value	**F** value	**p** value	**F** value	**p** value	**F** value	**p** value
CAR								
Model	36.47	<0.0001^∗^	8.91	0.0061^∗^	10.52	0.0037^∗^	1.13	0.4265
*A*-DT	45.87	0.0003^∗^	16.48	0.0048^∗^	17.73	0.0040^∗^	1.50	0.2605
*B*-COP	52.69	0.0002^∗^	6.09	0.0430^∗^	2.22	0.1801	1.84	0.2175
AB	13.48	0.0079^∗^	3.41	0.1073	2.34	0.1701	1.31	0.2901
*A* ^2^	12.89	0.0089^∗^	1.33	0.2861	0.72	0.4237	0.90	0.3756
*B* ^2^	63.71	<0.0001^∗^	18.20	0.0037^∗^	30.28	0.0009^∗^	0.18	0.6832
CCR								
Model	41.22	<0.0001^∗^	7.22	0.0109^∗^	12.47	0.0022^∗^	0.38	0.8501
*A*-DT	39.44	0.0004^∗^	6.46	0.0386^∗^	17.17	0.0043^∗^	1.52	0.2570
*B*-COP	45.69	0.0003^∗^	4.17	0.0805	5.19	0.0568	0.02	0.8866
AB	14.39	0.0068^∗^	3.04	0.1246	1.39	0.2764	<0.01	0.9870
*A* ^2^	5.90	0.0455^∗^	0.14	0.7223	0.28	0.6149	0.09	0.7729
*B* ^2^	105.39	<0.0001^∗^	22.37	0.0021^∗^	38.49	0.0004^∗^	0.28	0.6104
CAW								
Model	46.82	<0.0001^∗^	7.21	0.0110^∗^	8.82	0.0062^∗^	4.94	0.0296^∗^
*A*-DT	61.23	0.0001^∗^	15.90	0.0053^∗^	12.47	0.0096^∗^	3.69	0.0960
*B*-COP	67.66	<0.0001^∗^	2.18	0.1833	2.78	0.1393	15.15	0.0060^∗^
AB	4.90	0.0624	1.37	0.2807	0.47	0.5174	0.02	0.8952
*A* ^2^	21.74	0.0023^∗^	1.36	0.2820	1.61	0.2456	0.77	0.4084
*B* ^2^	88.29	<0.0001^∗^	16.21	0.0050^∗^	28.07	0.0011^∗^	5.51	0.0513
CCW								
Model	23.86	0.0003^∗^	11.49	0.0029^∗^	9.49	0.0051^∗^	0.65	0.6740
*A*-DT	44.24	0.0003^∗^	31.18	0.0008^∗^	17.60	0.0041^∗^	1.94	0.2068
*B*-COP	14.02	0.0072^∗^	2.95	0.1298	7.36	0.0301^∗^	1.06	0.3381
AB	3.21	0.1163^∗^	2.04	0.1962	1.23	0.3036	0.16	0.7009
*A* ^2^	14.45	0.0067^∗^	3.11	0.1213	1.32	0.2887	0.08	0.7869
*B* ^2^	49.37	0.0002^∗^	19.84	0.0030^∗^	20.93	0.0026^∗^	<0.01	0.9869

DT—drying temperature; COP—concentration of pretreatment; CAR—cassava from red landrace pretreated with citric acid; CCR—cassava from red landrace pretreated with calcium chloride; CAW—cassava from white landrace pretreated with citric acid; CCW—cassava from white landrace pretreated with calcium chloride; *A*—linear effect of DT; *B*—linear effect of COP; AB—interactive effect of DT and COP; *A*^2^—quadratic effect of DT; *B*^2^—quadratic effect of COP. ^∗^Significant at 95% confidence level.

**Table 3:** Regression models relating proximate components of cassava flour and model parameters.

Dependent variables	Models	*R* ^2^
	CAR	
Ash	+1.06 + 0.6651*A* + 0.7128*B* + 0.5100AB + 0.3781*A*^2^ + 0.8406*B*^2^	0.9630
Crude fibre	+3.08 + 0.4897*A* + 0.2976*B* + 0.3150AB + 0.1494*A*^2^ + 0.5519*B*^2^	0.8642
Moisture	+10.13 − 0.6962*A* − 0.2461*B* − 0.3575AB − 0.1506*A*^2^ − 0.9756*B*^2^	0.8825
Starch	+80.33 + 0.4879*A* + 0.5401*B* + 0.6450AB + 0.4044*A*^2^ + 0.1819*B*^2^	0.4459
	CCR	
Ash	+0.7900 + 0.5063*A* + 0.5450*B* + 0.4325AB + 0.2100*A*^2^ + 0.8875*B*^2^	0.9672
Crude fibre	+3.05 + 0.3684*A* + 0.2959*B* + 0.3575AB + 0.0575*A*^2^ + 0.7350*B*^2^	0.8376
Moisture	+10.14 − 0.6516*A* − 0.3582*B* − 0.2625AB − *A*^2^ − 1.05*B*^2^	0.8990
Starch	+79.18 + 0.5180*A* − 0.0621*B* − 0.0100AB + 0.1350*A*^2^ + 0.2400*B*^2^	0.2120
	CAW	
Ash	+1.11 + 0.6435*A* + 0.6765*B* + 0.2575AB + 0.4112*A*^2^ + 0.8288*B*^2^	0.9710
Crude fibre	+3.19 + 0.5005*A* + 0.1853*B* + 0.2075AB + 0.1569*A*^2^ + 0.5419*B*^2^	0.8375
Moisture	+10.20 − 0.6668*A* − 0.3157*B* − 0.1825AB − 0.2573*A*^2^ − 1.08*B*^2^	0.8631
Starch	+80.17 + 0.6714*A* + 1.36*B* + 0.0675AB + 0.3294*A*^2^ + 0.8794*B*^2^	0.7792
	CCW	
Ash	+0.9000 + 0.5578*A* + 0.3140*B* + 0.2125AB + 0.3419*A*^2^ + 0.6319*B*^2^	0.9446
Crude fibre	+3.18 + 0.4836*A* + 0.1487*B* + 0.1750AB + 0.1638*A*^2^ + 0.4138*B*^2^	0.8914
Moisture	+9.97 − 0.5879*A* − 0.3802*B* − 0.2200AB − 0.1725*A*^2^ − 0.6875*B*^2^	0.8714
Starch	+79.91 + 0.4301*A* + 0.3178*B* + 0.1750AB − 0.0931*A*^2^ − 0.0056*B*^2^	0.3158

CAR—cassava from red landrace pretreated with citric acid; CCR—cassava from red landrace pretreated with calcium chloride; CAW—cassava from white landrace pretreated with citric acid; CCW—cassava from white landrace pretreated with calcium chloride; *R*^2^—coefficient of determination; *A*—linear effect of drying temperature; *B*—linear effect of concentration of pretreatment; AB—interactive effect of drying temperature and concentration of pretreatment; *A*^2^—quadratic effect of drying temperature; *B*^2^—quadratic effect of concentration of pretreatment.

**Table 4:** Experimental runs and colour properties of cassava flour (CCR) under different processing conditions.

CCR	Independent variables		Dependent variables						
ER	DT (°C)	COP (%*w*/*v*)	*L* ^∗^	*a* ^∗^	*b* ^∗^	Chroma	WI	BI	Δ*E*
1	50	1	93.43 ± 0.01^c^	0.08 ± 0.01^b^	5.17 ± 0.05^d^	5.17 ± 0.05^d^	93.80 ± 0.01^a^	1.34 ± 0.01^cde^	2.37 ± 0.01^bc^
2	70	1	93.46 ± 0.09^c^	−0.25 ± 0.03^g^	5.84 ± 0.26^b^	5.84 ± 0.26^b^	92.57 ± 0.00^c^	1.46 ± 0.06^b^	2.15 ± 0.10^cd^
3	50	3	93.35 ± 0.09^c^	0.19 ± 0.02^a^	4.55 ± 0.15^f^	4.55 ± 0.15^e^	93.70 ± 0.09^a^	1.20 ± 0.04^g^	2.68 ± 0.04^a^
4	70	3	93.62 ± 0.03^bc^	−0.52 ± 0.02^h^	5.80 ± 0.08^b^	5.82 ± 0.08^b^	92.57 ± 0.00^c^	1.40 ± 0.02^c^	2.33 ± 0.05^c^
5	45	2	92.98 ± 0.09^d^	0.03 ± 0.03^c^	5.11 ± 0.22^de^	5.11 ± 0.22^d^	92.57 ± 0.00^c^	1.32 ± 0.06^de^	2.01 ± 0.19^d^
6	74	2	92.57 ± 0.41^e^	−0.10 ± 0.01^d^	5.41 ± 0.11^c^	5.41 ± 0.11^c^	93.03 ± 0.37^b^	1.39 ± 0.03^cd^	1.51 ± 0.37^e^
7	60	0.6	93.92 ± 0.02^a^	−0.28 ± 0.02^g^	5.50 ± 0.05^c^	5.51 ± 0.05^c^	92.57 ± 0.00^c^	1.36 ± 0.01^cde^	2.67 ± 0.03^a^
8	60	3.4	93.77 ± 0.04^ab^	−0.14 ± 0.03^e^	4.95 ± 0.08^de^	4.96 ± 0.08^d^	92.57 ± 0.00^c^	1.24 ± 0.02^fg^	2.75 ± 0.07^a^
9	60	2	93.54 ± 0.05^bc^	0.22 ± 0.03^a^	4.91 ± 0.10^e^	4.91 ± 0.10^d^	93.89 ± 0.05^a^	1.30 ± 0.02^ef^	2.61 ± 0.02^ab^
10	60	0	91.37 ± 0.16^f^	−0.02 ± 0.02^f^	6.30 ± 0.14^a^	6.31 ± 0.14^a^	91.99 ± 0.15^d^	1.63 ± 0.03^a^	—

ER—experimental run; DT—drying temperature; COP—concentration of pretreatment; CCR—cassava from red landrace pretreated with calcium chloride; *L*^∗^—lightness/darkness; *a*^∗^—redness/greenness; *b*^∗^—yellowness/blueness; WI—whiteness index; BI—brownness index; Δ*E*—colour difference. Values are mean ± standard deviation (*n* = 3). Means in the same column with different superscripts are significantly different at 95% confidence level.

**Table 5:** Experimental runs and colour properties of cassava flour (CCW) under different processing conditions.

CCW	Independent variables		Dependent variables						
ER	DT (°C)	COP (%*w*/*v*)	*L* ^∗^	*a* ^∗^	*b* ^∗^	Chroma	WI	BI	Δ*E*
1	50	1	92.98 ± 0.16^e^	0.11 ± 0.01^d^	5.88 ± 0.12^de^	5.88 ± 0.12^de^	93.43 ± 0.15^a^	1.55 ± 0.03^e^	2.21 ± 0.14^c^
2	70	1	92.75 ± 0.13^c^	0.02 ± 0.04^e^	6.15 ± 0.14^c^	6.15 ± 0.14^c^	92.57 ± 0.00^b^	1.61 ± 0.04^d^	1.87 ± 0.12^de^
3	50	3	93.29 ± 0.15^b^	0.55 ± 0.01^a^	4.78 ± 0.03^h^	4.78 ± 0.03^h^	93.66 ± 0.14^a^	1.33 ± 0.01^g^	3.26 ± 0.09^a^
4	70	3	92.78 ± 0.08^c^	−0.14 ± 0.03^g^	6.58 ± 0.10^b^	6.58 ± 0.10^b^	92.57 ± 0.00^b^	1.69 ± 0.03^c^	1.68 ± 0.11^e^
5	45	2	93.07 ± 0.06^bc^	0.23 ± 0.02^c^	5.03 ± 0.08^g^	5.03 ± 0.08^g^	92.57 ± 0.00^b^	1.34 ± 0.02^fg^	2.89 ± 0.09^b^
6	74	2	91.88 ± 0.34^d^	0.36 ± 0.04^b^	6.55 ± 0.12^b^	6.56 ± 0.12^b^	92.47 ± 0.30^b^	1.79 ± 0.03^b^	1.04 ± 0.31^f^
7	60	0.6	92.96 ± 0.05^bc^	−0.12 ± 0.01^g^	6.05 ± 0.05^cd^	6.05 ± 0.05^cd^	92.57 ± 0.00^b^	1.55 ± 0.01^e^	2.09 ± 0.04^cd^
8	60	3.4	93.65 ± 0.03^a^	−0.25 ± 0.02^h^	5.56 ± 0.06^f^	5.56 ± 0.06^f^	92.57 ± 0.00^b^	1.38 ± 0.12^f^	2.95 ± 0.03^b^
9	60	2	92.96 ± 0.13^bc^	0.54 ± 0.03^a^	5.80 ± 0.09^e^	5.83 ± 0.09^e^	93.41 ± 0.13^a^	1.61 ± 0.03^d^	2.32 ± 0.06^c^
10	60	0	91.37 ± 0.34^bc^	−0.06 ± 0.03^f^	7.26 ± 0.15^a^	7.26 ± 0.15^a^	92.07 ± 0.32^c^	1.92 ± 0.04^a^	—

ER—experimental run; DT—drying temperature; COP—concentration of pretreatment; CCW—cassava from white landrace pretreated with calcium chloride; *L*^∗^—lightness/darkness; *a*^∗^—redness/greenness; *b*^∗^—yellowness/blueness; WI—whiteness index; BI—brownness index; Δ*E*—total colour change. Values are mean ± standard deviation (*n* = 3). Means in the same column with different superscripts are significantly different at 95% confidence level.

**Table 6:** Regression models relating model parameters and colour properties of cassava flour.

Dependent variables	Models	*R* ^2^
	CAR	
*L* ^∗^	+92.33 + 0.0353*A* − 0.0679*B* + 0.1900AB − 0.1469*A*^2^ + 2.156*B*^2^	0.6328
*a* ^∗^	+0.9700 − 0.2147*A* + 0.0913*B* − 0.1200AB − 0.0962*A*^2^ − 0.3862*B*^2^	0.9334
*b* ^∗^	+6.68 + 0.3138*A* − 0.0449*B* + 0.1900AB + 0.0788*A*^2^ − 0.0162*B*^2^	0.6968
Chroma	+6.75 + 0.2912*A* − 0.0394*B* + 0.1775AB + 0.0681*A*^2^ − 0.0469*B*^2^	0.6784
WI	+92.89 − 0.0617*A* − 0.0950*B* + 0.1900AB − 0.0381*A*^2^ − 0.1206*B*^2^	0.4832
BI	+1.94 + 0.0446*A* + 0.0060*B* + 0.0250AB + 0.0063*A*^2^ − 0.0837*B*^2^	0.7884
Δ*E*	+1.56 + 0.0844*A* + 0.0084*B* + 0.1175AB − 0.0937*A*^2^ − 0.0062*B*^2^	0.4241
	CCR	
*L* ^∗^	+93.54 − 0.0350*A* − 0.0165*B* − 0.0600AB − 0.3438*A*^2^ + 0.1912*B*^2^	0.8888
*a* ^∗^	+0.2200 − 0.1470*A* + 0.0051*B* − 0.0957AB − 0.1330*A*^2^ − 0.2137*B*^2^	0.8420
*b* ^∗^	+4.91 + 0.2930*A* − 0.1797*B* + 0.1450AB + 0.1994*A*^2^ + 0.1819*B*^2^	0.8311
Chroma	+4.91 + 0.2955*A* − 0.1772*B* + 0.1500AB + 0.1994*A*^2^ + 0.1869*B*^2^	0.8294
WI	+93.89 − 0.2137*A* − 0.0125*B* + 0.0250AB − 0.4262*A*^2^ − 0.5412*B*^2^	0.6756
BI	+1.30 + 0.0524*A* − 0.0462*B* + 0.0200AB + 0.0331*A*^2^ + 0.0056*B*^2^	0.8697
Δ*E*	+2.61 − 0.1596*A* + 0.0754*B* − 0.0325AB − 0.3881*A*^2^ + 0.0869*B*^2^	0.9576
	CAW	
*L* ^∗^	+91.80 − 0.2968*A* + 0.0786*B* − 0.2475AB + 0.1265*A*^2^ + 0.2981*B*^2^	0.9850
*a* ^∗^	+1.12 − 0.1753*A* − 0.0347*B* + 0.0750AB − 0.1794*A*^2^ − 0.4919*B*^2^	0.9660
*b* ^∗^	+7.07 + 0.7643*A* + 0.0567*B* + 0.6125AB − 0.1706*A*^2^ + 0.02844*B*^2^	0.9361
Chroma	+7.16 + 0.7462*A* + 0.0542*B* + 0.6175AB − 0.1944*A*^2^ + 0.2406*B*^2^	0.9341
WI	+92.44 − 0.1697*A* + 0.0712*B* − 0.1425AB + 0.0781*A*^2^ + 0.1431*B*^2^	0.6464
BI	+2.09 + 0.1838*A* + 0.0090*B* + 0.1850AB − 0.0854*A*^2^ − 0.0232*B*^2^	0.9374
Δ*E*	+9.57 − 0.0291*A* + 0.0278*B* − 0.0200AB + 0.0244*A*^2^ + 0.0019*B*^2^	0.8870
	CCW	
*L* ^∗^	+95.96 − 0.3029*A* + 0.1645*B* − 0.0700AB − 0.2275*A*^2^ + 0.1875*B*^2^	0.9078
*a* ^∗^	+0.5400 − 0.0741*A* + 0.0130*B* − 0.1492AB − 0.1021*A*^2^ − 0.3429*B*^2^	0.8626
*b* ^∗^	+5.80 + 0.5275*A* − 0.1704*B* + 0.3825AB + 0.0075*A*^2^ + 0.0150*B*^2^	0.9981
Chroma	+5.83 + 0.5255*A* − 0.1666*B* + 0.3750AB − 0.0015*A*^2^ + 0.0035*B*^2^	0.9972
WI	+93.41 − 0.2614*A* + 0.0287*B* − 0.0575AB − 0.3169*A*^2^ − 0.2919*B*^2^	0.6450
BI	+1.61 + 0.1320*A* − 0.0476*B* + 0.0750AB − 0.0150*A*^2^ − 0.0650*B*^2^	0.9592
Δ*E*	+2.32 − 0.5670*A* + 0.2595*B* − 0.3100AB − 0.1744*A*^2^ + 0.1031*B*^2^	0.9803

CAR—cassava from red landrace pretreated with citric acid; CCR—cassava from red landrace pretreated with calcium chloride; CAW—cassava from white landrace pretreated with citric acid; CCW—cassava from white landrace pretreated with calcium chloride; *L*^∗^—lightness/darkness; *a*^∗^—redness/greenness; *b*^∗^—yellowness/blueness; WI—whiteness index; BI—brownness index; Δ*E*—colour difference; *A*—linear effect of drying temperature; *B*—linear effect of concentration of pretreatment; AB—interactive effect of drying temperature and concentration of pretreatment; *A*^2^—quadratic effect of drying temperature; *B*^2^—quadratic effect of concentration of pretreatment.

**Table 7:** Experimental runs and colour properties of cassava flour (CAR) under different processing conditions.

CAR	Independent variables		Dependent variables						
ER	DT (°C)	COP (%*w*/*v*)	*L* ^∗^	*a* ^∗^	*b* ^∗^	Chroma	WI	BI	Δ*E*
1	50	1	92.94 ± 0.11^a^	0.69 ± 0.03^c^	6.30 ± 0.12^d^	6.34 ± 0.12^d^	93.43 ± 0.10^a^	1.77 ± 0.03^de^	1.81 ± 0.10^a^
2	70	1	92.15 ± 0.40^bc^	0.34 ± 0.02^e^	6.85 ± 0.04^bc^	6.86 ± 0.04^bc^	92.57 ± 0.00^c^	1.87 ± 0.01^c^	1.11 ± 0.32^e^
3	50	3	92.14 ± 0.16^bc^	0.97 ± 0.01^a^	5.91 ± 0.04^e^	5.99 ± 0.04^e^	92.67 ± 0.14^c^	1.73 ± 0.01^e^	1.46 ± 0.09^c^
4	70	3	92.11 ± 0.14^bc^	0.14 ± 0.04^f^	7.22 ± 0.04^a^	7.22 ± 0.04^a^	92.57 ± 0.00^c^	1.93 ± 0.02^b^	1.23 ± 0.12^de^
5	45	2	91.91 ± 0.11^c^	0.92 ± 0.05^b^	6.78 ± 0.19^bc^	6.85 ± 0.20^bc^	92.57 ± 0.00^c^	1.97 ± 0.06^ab^	1.34 ± 0.06^cd^
6	74	2	92.29 ± 0.04^b^	0.54 ± 0.01^d^	7.24 ± 0.04^a^	7.26 ± 0.03^a^	92.90 ± 0.03^b^	2.01 ± 0.01^a^	1.52 ± 0.02^bc^
7	60	0.6	92.72 ± 0.05^a^	−0.08 ± 0.03^g^	6.94 ± 0.09^b^	6.94 ± 0.09^b^	92.57 ± 0.00^c^	1.80 ± 0.03^d^	1.50 ± 0.03^bc^
8	60	3.4	92.93 ± 0.07^a^	0.38 ± 0.02^e^	6.70 ± 0.03^c^	6.71 ± 0.03^c^	92.57 ± 0.00^c^	1.82 ± 0.01^cd^	1.71 ± 0.07^ab^
9	60	2	92.33 ± 0.09^b^	0.97 ± 0.05^a^	6.68 ± 0.10^c^	6.75 ± 0.10^c^	92.89 ± 0.08^b^	1.94 ± 0.04^b^	1.56 ± 0.03^bc^
10	60	0	91.37 ± 0.16^d^	−0.20 ± 0.02^h^	6.30 ± 0.14^d^	6.31 ± 0.14^d^	91.99 ± 0.15^d^	1.63 ± 0.03^f^	—

ER—experimental run; DT—drying temperature; COP—concentration of pretreatment; CAR—cassava from red landrace pretreated with citric acid; *L*^∗^—lightness/darkness; *a*^∗^—redness/greenness; *b*^∗^—yellowness/blueness; WI—whiteness index; BI—brownness index; Δ*E*—total colour change. Values are mean ± standard deviation (*n* = 3). Means in the same column with different superscripts are significantly different at 95% confidence level.

**Table 8:** Experimental runs and colour properties of cassava flour (CAW) under different processing conditions.

CAW	Independent variables		Dependent variables						
ER	DT (°C)	COP (%*w*/*v*)	*L* ^∗^	*a* ^∗^	*b* ^∗^	Chroma	WI	BI	Δ*E*
1	50	1	92.19 ± 0.06^bc^	0.88 ± 0.00^b^	6.95 ± 0.14^c^	7.00 ± 0.13^c^	92.78 ± 0.06^b^	1.99 ± 0.04^cd^	9.56 ± 0.00^cd^
2	70	1	92.12 ± 0.14^cd^	0.34 ± 0.15^d^	7.17 ± 0.16^c^	7.18 ± 0.15^bc^	92.57 ± 0.00^c^	1.96 ± 0.05^d^	9.54 ± 0.00^d^
3	50	3	92.91 ± 0.07^a^	0.57 ± 0.05^c^	5.57 ± 0.06^d^	5.60 ± 0.05^d^	93.35 ± 0.06^a^	1.55 ± 0.01^f^	9.69 ± 0.01^a^
4	70	3	91.85 ± 0.23^de^	0.33 ± 0.04^d^	8.24 ± 0.27^a^	8.25 ± 0.27^a^	92.57 ± 0.00^c^	2.26 ± 0.09^a^	9.59 ± 0.03^b^
5	45	2	92.45 ± 0.02^bc^	0.90 ± 0.02^b^	5.79 ± 0.07^d^	5.86 ± 0.07^d^	92.57 ± 0.00^c^	1.68 ± 0.02^e^	9.66 ± 0.01^a^
6	74	2	91.57 ± 0.08^ef^	0.46 ± 0.03^d^	8.07 ± 0.23^a^	8.08 ± 0.23^a^	92.31 ± 0.09^d^	2.24 ± 0.07^a^	9.58 ± 0.02^bc^
7	60	0.6	92.29 ± 0.17^bc^	0.04 ± 0.01^f^	7.57 ± 0.33^b^	7.57 ± 0.33^b^	92.57 ± 0.00^c^	2.01 ± 0.09^cd^	9.56 ± 0.01^cd^
8	60	3.4	92.42 ± 0.23^bc^	0.07 ± 0.06^f^	8.11 ± 0.42^a^	8.11 ± 0.42^a^	92.57 ± 0.00^c^	2.16 ± 0.13^ab^	9.59 ± 0.03^b^
9	60	2	91.80 ± 0.08^e^	1.12 ± 0.02^a^	7.07 ± 0.09^c^	7.16 ± 0.08^c^	92.44 ± 0.08^cd^	2.09 ± 0.02^bc^	9.57 ± 0.00^bcd^
10	60	0	91.37 ± 0.34^f^	−0.06 ± 0.03^g^	7.26 ± 0.15^bc^	7.26 ± 0.15^bc^	92.07 ± 0.32^e^	1.92 ± 0.04^d^	—

ER—experimental run; DT—drying temperature; COP—concentration of pretreatment; CAW—cassava from white landrace pretreated with citric acid; *L*^∗^—lightness/darkness; *a*^∗^—redness/greenness; *b*^∗^—yellowness/blueness; WI—whiteness index; BI—brownness index; Δ*E*—total colour change. Values are mean ± standard deviation (*n* = 3). Means in the same column with different superscripts are significantly different at 95% confidence level.

## Data Availability

Data underlying the findings in this study can be made available to readers on request. Request can be made to udoroelohor@yahoo.com.
